# High Prevalence of Colistin-Resistant Escherichia coli with Chromosomally Carried *mcr-1* in Healthy Residents in Vietnam

**DOI:** 10.1128/mSphere.00117-20

**Published:** 2020-03-04

**Authors:** Takahiro Yamaguchi, Ryuji Kawahara, Kouta Hamamoto, Itaru Hirai, Diep Thi Khong, Thang Nam Nguyen, Hoa Thi Tran, Daisuke Motooka, Shota Nakamura, Yoshimasa Yamamoto

**Affiliations:** a Department of Microbiology, Osaka Institute of Public Health, Osaka, Japan; b Laboratory of Microbiology, School of Health Sciences, University of the Ryukyus, Nishihara, Okinawa, Japan; c Center of Medical-Pharmaceutical Science and Technology Services, Thai Binh University of Medicine and Pharmacy, Thai Binh, Vietnam; d Genome Information Research Center, Research Institute for Microbial Diseases, Osaka University, Osaka, Japan; e Graduate School of Pharmaceutical Sciences, Osaka University, Osaka, Japan; f Life Science Research Center, Gifu University, Gifu, Japan; JMI Laboratories

**Keywords:** *Escherichia coli*, Vietnam, chromosomal *mcr*, colistin resistance, residents

## Abstract

The wide distribution of colistin-resistant bacteria in developing countries has become a common phenomenon. To understand the mechanisms underlying their distribution, we studied the *mcr* genetic background of colistin-resistant Escherichia coli isolates from the fecal microbiota of healthy human residents from a community in Vietnam with a high prevalence of colistin-resistant E. coli with *mcr*. Fifty-seven colistin-resistant isolates were obtained from 98 residents; one isolate was collected from each individual and analyzed for *mcr*. We found that 36.8% of the isolates carried chromosomal *mcr-1*. Further, 63.2% and 1.8% of the isolates carried *mcr-1* on the plasmid and the plasmid/chromosome, respectively. Whole-genome sequencing of genetically unrelated isolates showed that the majority (6 of 7) of the isolates had the chromosomal *mcr-1* in a complete ancestral *mcr-1* transposon Tn*6330*, IS*Apl1*-*mcr-1*-*PAP2*-IS*Apl1*, which was inserted at various positions on the chromosomes. In addition, the majority (87.5%) of Tn*6330* of *mcr-1*-carrying plasmids (*n* = 8) lacked both upstream and downstream IS*Apl1* transposons. The results obtained in this study indicate that plasmid-to-chromosomal transfer of *mcr-1* may have occurred recently in the fecal microbiota of the residents. Additionally, Tn*6330* on the chromosome may lose IS*Apl1* from the transposon during multiplication to gain a more stable *mcr-1* state on the chromosome. Stabilization of resistance by the chromosomal incorporation of *mcr-1* would be an additional challenge in combating the dissemination of resistant bacteria.

**IMPORTANCE** Elucidation of the mechanism of the wide dissemination of colistin-resistant bacteria in communities of developing countries is an urgent public health issue. In this study, we investigated the genetic background of the colistin resistance gene *mcr* in E. coli isolates from the fecal microbiota of healthy human residents living in a community in Vietnam with a high prevalence of colistin-resistant E. coli. Our study revealed for the first time, a surprisingly high percentage (36.8%) of colistin-resistant E. coli carrying chromosomal *mcr-1*, the emergence of which may have occurred recently, in the fecal microbiota of the community residents. The *mcr-1* transposon on the chromosome may develop into a more stable genotype by the loss of insertion sequences (ISs). Our results are valuable in understanding the mechanism underlying the increasing prevalence of colistin-resistant bacteria within a community.

## Abstract (precis)

Elucidation of the mechanism of the wide dissemination of colistin-resistant bacteria in communities of developing countries is an urgent public health issue. In this study, we investigated the genetic background of the colistin resistance gene *mcr* in E. coli isolates from the fecal microbiota of healthy human residents living in a community in Vietnam with a high prevalence of colistin-resistant E. coli. Our study revealed for the first time, a surprisingly high percentage (36.8%) of colistin-resistant E. coli carrying chromosomal *mcr-1*, the emergence of which may have occurred recently, in the fecal microbiota of the community residents. The *mcr-1* transposon on the chromosome may develop into a more stable genotype by the loss of insertion sequences (ISs). Our results are valuable in understanding the mechanism underlying the increasing prevalence of colistin-resistant bacteria within a community.

## INTRODUCTION

Colistin is recognized as the last-resort antibiotic for the treatment of infectious diseases caused by multidrug-resistant (MDR) Gram-negative bacteria, including carbapenem-resistant bacteria. However, the wide distribution of colistin-resistant (COR) bacteria threatens the effectiveness of treatments with colistin (1).

Current reports of a wide distribution of COR Escherichia coli with a mobile resistance gene, *mcr*, in a community highlight the importance of the stability and possible transfer of the resistance gene to the pathogen (2). In contrast to outbreaks in nosocomial settings, the wide distribution of COR bacteria that may be occurring over a long period in the community seems to be attributable to exposure to a persistent, low-concentration antibiotic pressure. This hypothesis is supported by the frequent use of colistin-containing feed in livestock (3) and the retention of COR E. coli in the fecal microbiota of livestock (4). However, in a previous study, we showed that the wide distribution of COR E. coli in residents was not due to the clonal distribution of a certain lineage of COR bacteria (2). Therefore, the transfer of the *mcr*-carrying plasmid is the most likely explanation for the wide distribution of the gene. In this regard, recent reports revealed that *mcr-1* could be mobilized as an IS*Apl1*-flanked composite transposon, Tn*6330* (5, 6). Therefore, the structure of the IS*Apl1* transposon with *mcr* potentially reflects the transposition of *mcr* in COR bacterial isolates in the community. However, further investigations are needed to confirm this hypothesis.

The long-term stability of COR bacteria in the community is another factor that affects their distribution. Chromosomal *mcr* may play an important role in their stability, but this has not been definitively established. To further understand the distribution mechanisms, in the present study, we investigated the *mcr* location, as well as the *mcr* transposon structure, of COR E. coli isolates obtained from healthy subjects in Vietnam residing in a community where COR bacteria are frequently detected.

## RESULTS AND DISCUSSION

### Location of *mcr-1* in COR E. coli isolates.

The location of *mcr-1* in 57 COR E. coli isolates that were originally obtained from 98 asymptomatic healthy residents of a rural community in Vietnam during a previous study (2) was assessed using S1/I-CeuI pulsed-field gel electrophoresis (PFGE) and Southern blot hybridization with *mcr-1* probe analysis. All isolates used in this study were phylogenetically diverse, as determined by PFGE and multilocus sequence typing (MLST) analyses (Table 1). In addition, no clonal expansion in the community was observed, as determined in a previous study (2).

**TABLE 1:**
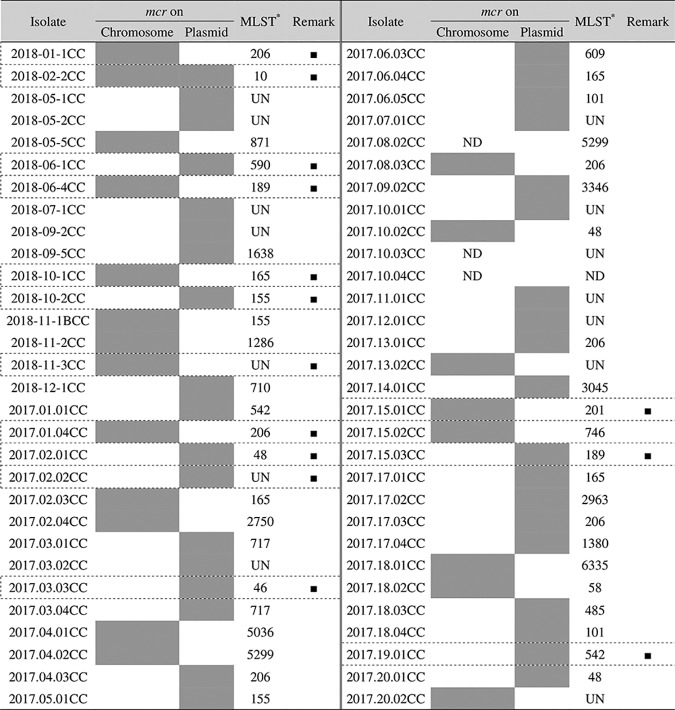
Location of *mcr-1* in colistin-resistant Escherichia coli isolates tested^*a*^

a Gray boxes indicate the locations where *mcr* was detected. ■, genome analysis was conducted; ND, not done; UN, unknown; *, data from the work of Yamamoto et al. (2).

Southern blot analysis of the isolates after S1-PFGE (see Fig. S1 in the supplemental material) revealed that 36.8% (21/57) of the isolates carried *mcr-1* on their chromosomes, whereas 63.2% (36/57) carried *mcr-1* on only the plasmid. (Three of the original 60 *mcr-1*^+^ E. coli isolates, from 98 residents, were excluded because of the difficulty in analysis.) In one isolate, *mcr-1* was located on both the chromosome and the plasmid.

10.1128/mSphere.00117-20.1 FIG S1 Representative results of Southern blot hybridization after S1 pulsed-field gel electrophoresis with *mcr-1* and 16S rRNA probes. Row numbers indicate isolates as follows: 1, 2017.01-1CC; 2, 2018-16-2CC; 3, 2018-12-1CC; 4*, 2018-11-3CC; 5, 2018-11-2CC; 6, 2018-11-1BCC; 7, 2018-10-3BCC; 8, 2018-10-2CC; 9*, 2018-10-1CC; 10, 2018-09-5CC; 11, 2018-09-2CC; 12, 2018-08-1CC; M, marker. The asterisk indicates the isolate for which genome analysis was performed. The arrow indicates the specific probe binding position. Download FIG S1, PDF file, 0.03 MB. Copyright © 2020 Yamaguchi et al. 2020 Yamaguchi et al. This content is distributed under the terms of the Creative Commons Attribution 4.0 International license.

*mcr-1* was originally discovered as a mobile resistance gene on the plasmid of COR bacteria (7), and it is recognized that chromosomally carried *mcr-1* is very rare compared to that on the plasmids (8–13). For instance, Li et al. found that only 4% of 200 *mcr-1*-positive E. coli isolates from animals, food, the environment, and human fecal samples collected in China carried chromosomal *mcr-1* (11). In contrast to previous reports, the results from this study showed that 36.8% of the human fecal sample isolates had chromosomal *mcr-1*. All isolates examined in this study were obtained from different individuals, except two isolates that were obtained from the same person at a 1-year interval. The prevalence of chromosomal *mcr-1* observed in this study is extremely high compared to that reported in previous studies. Although the reasons for the high prevalence of chromosomal *mcr-1*-carrying isolates in human fecal microbiota are not clear, it seems likely that the high prevalence in the area of sampling could be due to the frequent use of colistin as a livestock antibiotic and feed additive (3). Additionally, most domestic livestock in that community possessed COR E. coli carrying *mcr-1* (4). Under the prevailing conditions, it is conceivable that the community from which the isolates were obtained could have experienced a constant colistin pressure on the microbes over a long period, which resulted in the microbes becoming intrinsically resistant due to chromosomally encoded resistance (11). This could have resulted in chromosomal *mcr-1* becoming more prevalent and stable in the community.

The chromosomal *mcr-1*-carrying bacteria may contribute to the emergence of untreatable MDR bacteria when MDR genes, including carbapenem resistance genes on the plasmid (14), are transferred to bacteria possessing chromosomal *mcr*. In fact, this likelihood is supported by our current study, wherein several extended-spectrum β-lactamase (ESBL)-producing E. coli isolates that had MDR with *mcr-1* were obtained from fecal microbiota of healthy human residents of the community (15).

### Genetic structure of chromosomally and/or plasmid-carried *mcr-1* transposon.

Recently, it has been proposed that *mcr-1* can be mobilized as an IS*Apl1*-flanked composite transposon (Tn*6330*) (5, 16). The study also reported that transmission is possible both upstream and downstream of IS*Apl1*. The structure of this composite transposon is considered to be stabilized by the structure in which insertion sequences (ISs) have dropped out (6, 17). Therefore, the structure of *mcr-1* transposon Tn*6330* in the bacterium is important not only for the transmission among microbes but also for the stability of *mcr-1*.

To elucidate the *mcr-1* transposon structure of the isolates in this study, 14 representative strains (6 of 18 chromosomal *mcr-1* isolates, one chromosomal/plasmid *mcr-1* isolates, and 7 of 30 plasmid *mcr-1* isolates) were subjected to genome analysis. Several strains were obtained from the same household (Table 2). In the case of household 2, two *mcr-1* isolates were obtained from the same member at a 1-year interval. All these isolates were phylogenetically different (2). Table 2 shows the bacterial characteristics of chromosomally and/or plasmid-carried *mcr-1* transposons for these 14 isolates.

**TABLE 2:** Characterization of chromosomally and/or plasmid-carried *mcr-1* transposon of colistin-resistant Escherichia coli

Household	Household member	Yr of isolation	Isolate	MLST type^*a*^	No. of plasmid	*mcr* carriage	Size (kbp)^*a*^	*mcr* location (kbp)	*mcr* transposon	Transposon type^*b*^	Plasmid Inc type
1	A	2017	2017.01.04CC	ST206	2	Chromosome	4,615	3215	IS*Apl1*-*mcr1*- *PAP2*-IS*Apl1*	A	
	B	2018	2018-01-1CC	ST206	3	Chromosome	4,711	1754	IS*Apl1*-*mcr1*- *PAP2*-IS*Apl1*	A	

2	A	2017	2017.02.01CC	ST48	4	Plasmid^*e*^	231		*mcr1*-*PAP2*	C	IncHI2
	B	2017	2017.02.02CC	UN^*c*^	4	Plasmid	34		*mcr1*-*PAP2*	C	IncX4
		2018	2018-02-2CC	ST10	3	Chromosome	4,574	1271	IS*Apl1*-*mcr1*-*PAP2*	B	
						Plasmid	47		*mcr1*-*PAP2*	C	IncP1

3	A	2017	2017.03.03CC	ST46	4	Plasmid	294		*mcr1*-*PAP2*	C	IncHI2

6	A	2018	2018-06-4CC	ST189	4	Chromosome	4,753	3569	IS*Apl1*-*mcr1*- *PAP2*-IS*Apl1*	A	
	B	2018	2018-06-1CC	ST590	4	Plasmid	33		*mcr1*-*PAP2*	C	IncX4

10	A	2018	2018-10-1CC	ST165	4	Chromosome	4,701	923	IS*Apl1*-*mcr1*- *PAP2*-IS*Apl1*	A	
	B	2018	2018-10-2CC	ST155	2	Plasmid	60		*mcr1*-*PAP2*	C	IncI2

11	A	2018	2018-11-3CC	ST206-like^*d*^	1	Chromosome	4,557	1449	IS*Apl1*-*mcr1*- *PAP2*-IS*Apl1*	A	
											
15	A	2017	2017.15.01CC	ST201	4	Chromosome	4,869	2670	IS*Apl1*-*mcr1*- *PAP2-*IS*Apl1*	A	
	B	2017	2017.15.03CC	ST189	3	Plasmid	104		IS*Apl1*-*mcr1*-*PAP2*	B	IncY

19	A	2017	2017.19.01CC	ST542	10	Plasmid	33		*mcr1*-*PAP2*	C	IncX4

a Size of chromosome or plasmid.

b Data from work of Snesrud et al. (6).

c UN, unknown.

d Only *adk* was different, compared to ST206.

e Plasmids with similar levels of shading are similar to one another.

### Plasmid-carried *mcr-1* transposon.

Two isolates obtained from different residents showed different host bacterium MLST types but possessed very similar IncHI2 plasmids with the same *mcr-1* transposon, *mcr-1*-*PAP2*. Similarly, three other isolates showed different E. coli MLST types but had the same IncX4 plasmid with the *mcr-1*-*PAP2* transposon (Table 2). These results indicate that the transfer of plasmids among bacteria may occur frequently in the human bacterial flora.

Other plasmids showed different *mcr-1* transposons, including IS*Apl1*-*mcr-1*-*PAP2*, which was carried by different Inc types, such as IncP1, IncI2, and IncY. These results confirmed that *mcr-1* was retained in various Inc-type plasmids in bacteria from fecal microbiota of residents in the community, as previously shown by the diversity of *mcr-1*-carrying plasmids (9). In addition, the majority (7 of 8) of plasmids that were assessed had the simplest *mcr-1* transposon structure, *mcr-1*-*PAP2*. One remaining plasmid had the IS*Apl1*-*mcr-1*-*PAP2* transposon. Our results indicate that *mcr-1* on the plasmid of COR E. coli strains in the fecal microbiota of humans may be stabilized by the loss of IS*Apl1* in many cases, as described by Wang et al. (17).

### Chromosomally carried *mcr-1* transposon Tn*6330*.

**FIG 1:**
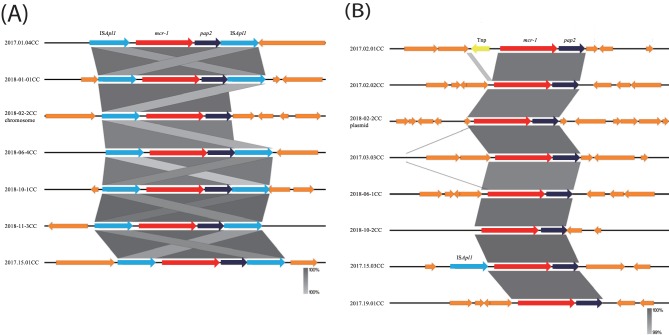
Comparison of the genetic structures of the *mcr-1* transposon in Escherichia coli isolates. (A) Isolates possessing chromosomal *mcr-1*. (B) Isolates possessing plasmid *mcr-1*.

The *mcr-1* transposon structure in the chromosome of the seven COR E. coli isolates assessed in this study showed that the majority (6 of 7) of the isolates possessed a complete ancestral *mcr-1* transposon, Tn*6330* (6), without the loss of IS*Apl1* in their chromosomes (Fig. 1). The Tn*6330* insertion sites on the chromosomes of these isolates were random. Besides, the AT- and CG-rich sequences at either end of Tn*6330* were not found on the chromosomes of the isolates (data not shown). Because the insertion sites of Tn*6330* are notable for AT- and CG-rich regions (6), it is presumable that the absence of such 2-bp target site duplications in these isolates may be caused by mutations after the transposition of Tn*6330*, even though there was no evidence to support it. The one remaining isolate lost the IS*Apl1* present downstream of Tn*6330*, which may contribute to stabilizing the *mcr-1* gene (16).

The genome analysis of these isolates revealed that there was only one Tn*6330* in the chromosome of the isolates tested. In addition, there was no independent *mcr-1* without an IS in the chromosomes. Thus, the results from the genome assessment of COR E. coli isolates of fecal microbiota from the community residents support a previous finding that IS*Apl1* facilitates *mcr-1* transmission (11).

The dominant fully intact Tn*6330*, IS*Apl1*-*mcr-1*-*PAP2*-IS*Apl1*, on the chromosome among COR E. coli isolates from fecal microbiota of community residents may indicate that this transposon insertion into the chromosome occurred recently. If the high prevalence of chromosomally carried *mcr-1* has occurred recently, it may indicate that the wide distribution of COR bacteria in the community has progressed to a stable state. Furthermore, although it was found in one isolate, the loss of IS*Apl1* from the *mcr-1* transposon Tn*6330* indicates that the resistance gene on the chromosome has shifted to a more stable state.

One isolate was found to harbor *mcr-1* on both the chromosome and plasmid. Because the origin of the *mcr-1* transposon is unclear from the transposon elements, the relationship between *mcr-1* on the chromosome and the plasmid in this isolate is unknown. In this regard, the following can be speculated. Because truncated Tn*6330* was found in all plasmids assessed in this study, it is likely that COR E. coli isolates with plasmids harboring Tn6*330* may have prevailed in the community for a long time. Moreover, it can be presumed that the transposition of Tn*6330* from the plasmid to the chromosome occurred, followed by the loss of the IS during the process of stabilizing *mcr-1*. Therefore, this isolate may be a transitional intermediate-type strain. The finding of the intermediate-type isolate suggests that the process for stabilizing *mcr-1* is in progress.

The assessment of *mcr-1* location in COR E. coli isolates in this study showed that only one isolate carried it both on the chromosome and on the plasmid, whereas the other isolates carried the *mcr-1* either on the chromosome or on the plasmid. It is not clear why the Tn*6330* was carried on either the plasmid or the chromosome and only rarely on both. Because large antibiotic-resistant plasmids may be lost during their multiplication in an antibiotic-free environment due to their significant metabolic burden on the host strain, it can be speculated that after the transposition of Tn*6330* from the plasmid to the chromosome, the plasmid may no longer need to carry Tn*6330* or the plasmid itself may not be needed (18).

## MATERIALS AND METHODS

### Sample collection for COR E. coli isolates.

A total of 57 COR E. coli isolates with *mcr-1* were assessed in this study. All the COR E. coli isolates were initially obtained from healthy residents in a rural community in Vietnam between November 2017 and February 2018. One isolate was obtained from each resident. It was found that a high percentage (70.4%) of the residents were carrying COR E. coli with *mcr-1* in their stool, as reported in a previous study (2).

### Assessment of *mcr-1*.

Digoxigenin (DIG)-labeled DNA probes used for the detection of *mcr-1* and 16S rRNA genes in Southern blot hybridization were prepared using the PCR DIG probe synthesis kit (Sigma-Aldrich, St. Louis, MO) with the primers shown in Table S1 in the supplemental material. The location of *mcr-1* in the examined isolates was determined by S1 nuclease PFGE and Southern blot hybridization, as previously described (19). For some bacterial isolates, I-CeuI PFGE and Southern blot hybridization were also performed to determine *mcr-1* locations according to the methods described in previous studies (20, 21).

10.1128/mSphere.00117-20.2 TABLE S1 Primers used for amplifying the probes for Southern blot hybridization. Download Table S1, DOCX file, 0.01 MB. Copyright © 2020 Yamaguchi et al. 2020 Yamaguchi et al. This content is distributed under the terms of the Creative Commons Attribution 4.0 International license.

### Genome sequencing.

Of the 57 COR E. coli isolates with *mcr*, a total of 14 isolates were chosen for genome sequencing (Table 1). Whole-genome sequencing and assembly of isolates, along with a plasmid harboring *mcr-1*, were performed on the Illumina MiSeq (Illumina Inc., CA) and MinION (Oxford Nanopore Technologies, London, United Kingdom) sequencers, as described previously (22). The genomes were annotated using DDBJ Fast Annotation and Submission Tool pipeline (https://dfast.nig.ac.jp). Genome analysis was performed using the Geneious R11 software (Biomatters, Ltd., Auckland, New Zealand), Easyfig (23), and BRIG (24).

### Accession number(s).

The draft genome sequences of the colistin-resistant E. coli strains 2017.01.04CC (chromosome), 2018-01-1CC (chromosome), 2017.02.01CC (plasmid), 2017.02.02CC (plasmid), 2018.02.2CC (chromosome and plasmid), 2017.03.03CC (plasmid), 2018-06-4CC (chromosome), 2018-06-1CC (plasmid), 2018-10-1CC (chromosome), 2018-10-2CC (plasmid), 2018-11-3CC (chromosome), 2017.15.01CC (chromosome), 2017.15.03CC (plasmid), and 2017.19.01CC (plasmid) were deposited in DDBJ/GenBank under the accession numbers AP021891, AP021892, LC511657, LC511656, AP021896 (chromosome), AP021897 (chromosome), LC511658, AP021893, LC511661, AP021894, LC511662, AP021895, AP021890, LC511659, and LC511660, respectively.
