# Supplementary figures and images for: High Prevalence of Colistin-Resistant Escherichia coli with Chromosomally Carried mcr-1 in Healthy Residents in Vietnam
Source: mSphere. 2020 Mar 4;5(2):e00117-20. doi: 10.1128/mSphere.00117-20 (PMC7056805; doi:10.1128/mSphere.00117-20)

(A) *mcr-1* probe

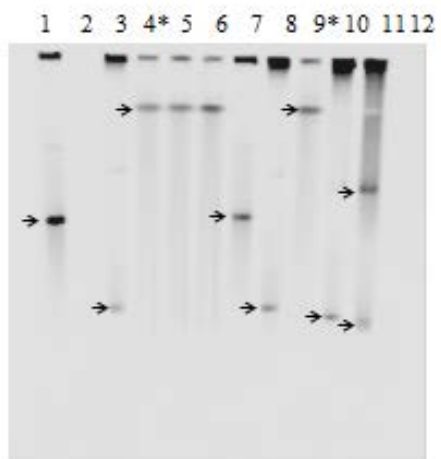

(B) 16S rRNA gene probe

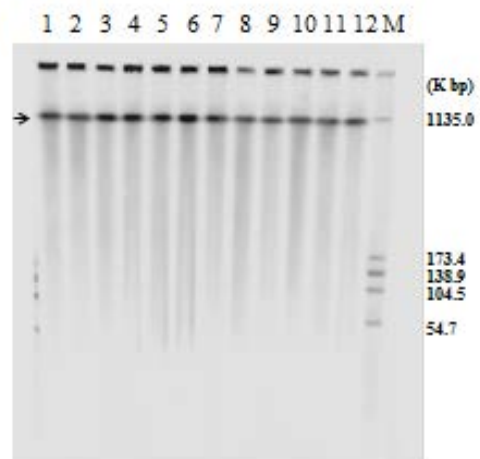

Supplement: FIG S1 [file mSphere.00117-20-sf001.pdf]
