# Supplementary material for: High Prevalence of Colistin-Resistant Escherichia coli with Chromosomally Carried mcr-1 in Healthy Residents in Vietnam
Source: mSphere. 2020 Mar 4;5(2):e00117-20. doi: 10.1128/mSphere.00117-20 (PMC7056805; doi:10.1128/mSphere.00117-20)
Supplement: TABLE S1 [file mSphere.00117-20-st001.docx]

| Primer name | Sequence (5′ – 3′) | Reference |
| --- | --- | --- |
| mcr-1 F | CGGTCAGTCCGTTTGTTC | 1 |
| mcr-1 R | AGCGATACTCATCTCAGCAAGT | This study |
| 16S rRNA gene F | CCAGCAGCCGCGGTAATACG | 2 |
| 16S rRNA gene R | ATCGGYTACCTTGTTACGACTTC | 2 |

1. Liu YY, Wang Y, Walsh TR, Yi LX, Zhang R, Spencer J, Doi Y, Tian G, Dong B, Huang X, Yu LF, Gu D, Ren H, Chen X, Lv L, He D, Zhou H, Liang Z, Liu JH, Shen J. 2016. Emergence of plasmid-mediated colistin resistance mechanism MCR-1 in animals and human beings in China: a microbiological and molecular biological study. Lancet Infect Dis 16:161-168. <https://doi.org/10.1016/S1473-3099(15)00424-7>.

2. Lu JJ, Perng CL, Lee SY, Wan CC. 2000. Use of PCR with universal primers and restriction endonuclease digestions for detection and identification of common bacterial pathogens in cerebrospinal fluid. J Clin Microbiol 38:2076-2080.
